# Prevalence of genetic variants of keratins 8 and 18 in patients with drug-induced liver injury

**DOI:** 10.1186/s12916-015-0418-0

**Published:** 2015-08-19

**Authors:** Valentyn Usachov, Thomas J. Urban, Robert J. Fontana, Annika Gross, Sapna Iyer, M. Bishr Omary, Pavel Strnad, on behalf of the Drug-Induced Liver Injury Network

**Affiliations:** Department of Internal Medicine III and IZKF, University Hospital Aachen, RWTH Aachen, Pauwelsstrasse 30, D-52074 Aachen, Germany; Division of Pharmacotherapy and Experimental Therapeutics, Center for Pharmacogenomics and Individualized Therapy, UNC Eshelman School of Pharmacy, UNC Hamner Institute for Drug Safety Sciences, University of North Carolina, Chapel Hill, NC USA; Department of Internal Medicine, University of Michigan Health System, Ann Arbor, MI USA; Department of Molecular and Integrative Physiology, University of Michigan Medical School, Ann Arbor, MI USA; Department of Internal Medicine I, University Medical Center Ulm, Ulm, Germany

**Keywords:** Intermediate filaments, Mutation, Drug-induced liver injury, DILI

## Abstract

**Background:**

Keratin 8 and 18 (K8/K18) cytoskeletal proteins protect hepatocytes from undergoing apoptosis and their mutations predispose to adverse outcomes in acute liver failure (ALF). All known K8/K18 variants occur at relatively non-conserved residues and do not cause keratin cytoskeleton reorganization, whereas epidermal keratin-conserved residue mutations disrupt the keratin cytoskeleton and cause severe skin disease. The aim of our study was to identify keratin variants in idiosyncratic drug-induced liver injury (DILI).

**Methods:**

Genomic DNA was isolated from 800 patients enrolled in an ongoing US multicenter study, with DILI attributed to a wide range of drugs. Specific K8/K18 exonic regions were PCR-amplified and screened by denaturing HPLC followed by DNA sequencing. The functional impact of keratin variants was assessed using cell transfection and immune staining.

**Results:**

Heterozygous and compound amino acid-altering K8/K18 variants were identified in 86 DILI patients and non-coding variants in 15 subjects. Five novel amino acid-altering (K8 Lys393Arg, K8 Ala351Val, K8 Ala358Val, K8 Ile346Val, K18 Asp89His) and two non-coding variants were observed. Several variants segregated with specific ethnic backgrounds but were found at similar frequencies in DILI subjects and ethnically matched population controls. Notably, variants in highly conserved residues of K8 Lys393Arg (ezetimibe/simvastatin-related) and K18 Asp89His (isoniazid-related) were found in patients with fatal DILI. These novel variants also led to keratin network disruption in transfected cells.

**Conclusions:**

Novel K8/K18 cytoskeleton-disrupting variants were identified in two patients and segregated with fatal DILI. Other non-cytoskeleton-disrupting keratin variants did not preferentially associate with DILI.

**Electronic supplementary material:**

The online version of this article (doi:10.1186/s12916-015-0418-0) contains supplementary material, which is available to authorized users.

## Background

Keratins (Ks) represent a subgroup of intermediate filaments (IFs) found mainly in epithelial tissues [1–3]. As for all IFs, they display a tripartite structure containing a conserved α-helical central rod domain that is flanked by a less conserved N-terminal head and C-terminal tail domains [4, 5]. Keratins are further subdivided into the relatively acidic type I (including K9–K28 and K31–K40) and relatively basic type II IFs (including K1–K8 and K71–K86) that associate as obligate non-covalent heteropolymers [6, 7]. Consequently, every cell type contains a characteristic type I–II expression pattern [1, 7]. For example, K1/K10 and K5/K14 are the major keratins of suprabasal and basal keratinocytes, respectively, while K8/K18/K19/K20 are the keratins found in simple type glandular epithelia [2, 8]. Adult hepatocytes are unique in that they only contain K8/K18, while most other cell types display a more complex keratin expression pattern [8, 9]. It is this heteropolymeric keratin–keratin association that accounts for the amino acid position-selective dominant negative effect of heterozygous keratin mutations in human disease.

Keratins are established cytoprotective proteins [10, 11] and keratin mutations lead or predispose to the development of >60 distinct human diseases [2, 12, 13] that are faithfully reproduced in transgenic animals models [8, 14]. In mouse liver, mutations or loss of K8/K18 cause a mild phenotype under basal conditions, but predispose to significant injury from a variety of stresses including apoptotic, metabolic, oxidative and drug-induced [11, 15–17]. Human association studies have identified keratin variants to be overrepresented in patients with end-stage liver disease, acute liver failure (ALF), chronic hepatitis C virus infection or primary biliary cirrhosis, and to predispose to adverse clinical outcomes [8, 18, 19].

Human studies also revealed marked differences in ethnic distribution of keratin variants. For example, K8 G62C and K8 R341H were the most abundant amino acid substitutions in Caucasians, while African-American (AA) subjects displayed high frequencies of K8 A333A and G434S variants [19]. Importantly, K8/K18 variants were found in the less conserved protein regions and did not affect keratin organization under basal conditions, while disease-causing mutations in epidermal keratins cluster in the most conserved subdomains at the amino and carboxyl regions of the rod domain, and often result in keratin network breakdown [2, 8, 14, 20]. For example, K14 R125C leads to the most severe Dowling Meara form of epidermolysis bullosa simplex (EBS) [14]. The homologous K18 alteration (K18 R90C) that was engineered in transgenic animals results in a disruption of filamentous hepatocellular keratin network, mild chronic hepatitis and distinct susceptibility to various hepatotoxic stresses [8, 15].

Given the murine data suggesting keratin mutation-associated susceptibility to chemical hepatotoxicity and human studies linking K8/K18 variants to acetaminophen hepatotoxicity [19], we hypothesized that K8/K18 variants may contribute to the susceptibility to human idiosyncratic drug-induced liver injury (DILI) that is a prominent cause of ALF [21, 22]. DILI is also a major adverse event leading to termination of clinical drug development programs [23]. Several previous studies suggested that DILI has a genetic component with variants in human leukocyte antigens and drug metabolizing enzymes increasing risk for development of DILI [22, 24]. We tested our hypothesis by analyzing 800 consecutive DILI subjects enrolled in the ongoing DILI Network (DILIN) study and identified several novel K8/K18 variants.

## Methods

### Study participants

Between August 2004 and April 2009, 808 subjects were recruited from eight US clinical sites participating in the DILIN. Details of the inclusion/exclusion criteria were previously described [25]. DILI causality assessment and severity was based on consensus of a panel of experienced hepatologists [25]. The cases were subdivided into hepatocellular, cholestatic or mixed injury pattern based on the R-value [25, 26]. All participants provided written informed consent, which included collection of DNA for genetic association studies, and the study was approved by the local institutional review boards of the participating centers (see list in the Acknowledgements section). For control data of keratin variants in Caucasian and African-American subjects, we used the Exome Sequencing Project database available through the Exome Variant Server [27] whereas the 1000 Genomes project data [28] were employed for Hispanic and Asian controls.

### Genetic analysis

Genomic DNA was obtained from EDTA-anticoagulated peripheral blood with a DNeasy Tissue Kit (Qiagen, Valencia, CA, USA). The mutational K8/K18 hotspots (K8 exons 1, 6, 8 (corresponding to amino acids 1–108, 328–400, 421–483); K18 exon 1 (corresponding to amino acids 1–139) and their adjacent exon-intron boundaries) were PCR-amplified using AmpliTaq Gold® DNA Polymerase (Applied Biosystems, Foster City, CA, USA) and previously described primers [29]. The selection of these hotspots is based on more than 2,000 human subjects in whom the entire K8/K18 exonic regions were analyzed [17–19, 29, 30]. All PCR products were pre-screened with the WAVE*®* denaturing HPLC system (Transgenomic, Omaha, NE, USA). Specimens with an abnormal elution peak were confirmed in an independent PCR analysis, purified and subjected to bidirectional DNA sequencing. Annotation of coding K8/K18 variants was made with the mRNA sequences NM_002273.3/NM_000224.2, while the sequences M34482.1/AF179904.1 were used for non-coding changes. The conservation of the observed K8/K18 variants was analyzed using the following sequences: K8: NP_002264.1 (human), NP_112447.2 (mouse), NP_001028782.1 (cow), NP_001080525.1 (frog), NP_956374.1 (zebrafish); K1 (NP_006112.3), K2 (NP_000414.2), K3 (NP_476429.2), K4 (NP_002263.2), K5 (NP_000415.2), K6a (NP_005545.1), K7 (NP_005547.3); K18: NP_000215.1 (human), NP_034794.2 (mouse), NP_001179024.1 (cow), NP_001089734.1 (frog), NP_848524.1 (zebrafish); K9 (NP_000217.2), K10 (NP_000412.3), K12 (NP_000214.1), K13 (NP_705694.2), K14 (NP_000517.2), K15 (NP_002266.2), K16 (NP_005548.2), K17 (NP_000413.1), K19 (NP_002267.2), K20 (NP_061883.1). Given that eight samples were not reliably amplified, 800 samples were included in the final analysis.

### Statistical analysis

The Fisher’s exact test was used to determine non-random associations between two variables, and *P* values less than 0.05 were considered statistically significant.

### Cell culture experiments

To study the biological significance of novel K8/K18 variants, human K8 and K18 cDNA inserted in the pcDNA3.1 vector was modified with the QuikChange® Site-Directed Mutagenesis Kit (Stratagene, Santa Clara, CA, USA) and specific primers (hK8-I346V-F ctgggagagctggccgttaaggatgccaacg, hK8-I346V-R cgttggcatccttaacggccagctctccacg; hK8-A351V-F cattaaggatgccaacgtcaagttgtccgagctgg, hK8-A351V-R ccagctcggacaacttgacgttggcatccttaatg; hK8-A358V-F cgagctggaggtcgccctgcagc, hK8-A358V-R gctgcagggcgacctccagctcg; hK8-K393R-F cgccacctacaggaggctgctggaggg, hK8-K393R-R ccctccagcagcctcctgtaggtggcg; hK18-D89H-F gcaaagcctgaaccaccgcctggcctc, hK18-D89H-R gaggccaggcggtggttcaggctttgc). The resulting constructs were verified by DNA sequencing. For immunofluorescence staining, NIH 3T3 cells (CRL-1658; American Type Culture Collection) were grown in DMEM medium (Gibco, Life Technologies GmbH, Darmstadt, Germany) supplemented with 10 % FCS, 1 % penicillin-streptomycin and 1 % L-glutamine, and then transfected with equal amounts of K8 or K18 variant cDNA together with an equal amount of non-mutated partner keratin (K18 or K8) cDNA using Lipofectamine 2000 (Invitrogen, Life Technologies GmbH, Darmstadt, Germany). Transfection of wild-type K8/K18 was used as control. Transfected cells were fixed with precooled −20 °C methanol (3 min) and acetone (15 s) after 24 hours, washed in PBS, and incubated with the anti-K18 antibody Ks 18.04 (Progen Biotechnik GmbH, Heidelberg, Germany) [31]. After washing and exposure to the secondary antibody, the glass slides were mounted in ProLong® Gold antifade reagent with DAPI mounting medium (Life Technologies Corporation, Eugene, OR, USA). To quantify the percentage of disrupted cells, all transfections were performed in triplicate and at least 100 cells were scored in each case. Cells were characterized as having normal-appearing or disrupted cytoskeletal keratin network. Coomassie staining for total protein lysates of transient transfected cells has shown equal levels of proteins. Of note, the transfection efficiency was similar in all subgroups and ranged between 50–70 %.

## Results

To address the importance of K8/K18 variants in DILI, 800 well characterized DILI subjects were analyzed. Of the examined subjects, 72 % were Caucasians, while Hispanics and African-Americans each constituted 11 % of the study cohort (Table 1). In 63 % of patients, DILI was deemed to be very likely or definite, while <5 % were scored as unlikely. Nearly 55 % of subjects had hepatocellular injury at presentation, while 25 % and 20 % displayed a cholestatic and mixed damage pattern, respectively. Fatal DILI was recorded in 9 % of patients and 55 % of participants required hospitalization due to their liver injury (Table 1).

**Table 1:** Characteristics of the DILI cohort

Gender	Male	Female	Total		
	335 (41.9)	465 (58.1)	800 (100)		
Ethnicity/race	AA	Asian	Caucasian	Hispanic	Other
	87 (10.9)	25 (3.1)	577 (72.1)	88 (11.0)	23 (2.9)
Causality^a^	Definitive	Very likely	Probable	Possible	Unlikely
	190 (23.8)	312 (39.0)	148 (18.5)	110 (13.8)	39 (4.9)
Injury type	Hepatocellular	Cholestatic	Mixed		
	438 (54.8)	198 (24.7)	164 (20.5)		
Severity	Fatal (5)	Severe (4)	Mod hosp (3)	Mod (2)	Mild (1)
	71 (8.9)	144 (18.0)	221 (27.6)	176 (22.0)	188 (23.5)

Absolute numbers are shown followed by percentages in brackets. ^a^Causality score was unknown in one patient. AA, African-American; DILI, drug-induced liver injury; Mod, moderate non-hospitalized; Mod hosp, moderate hospitalized

**Fig. 1:**
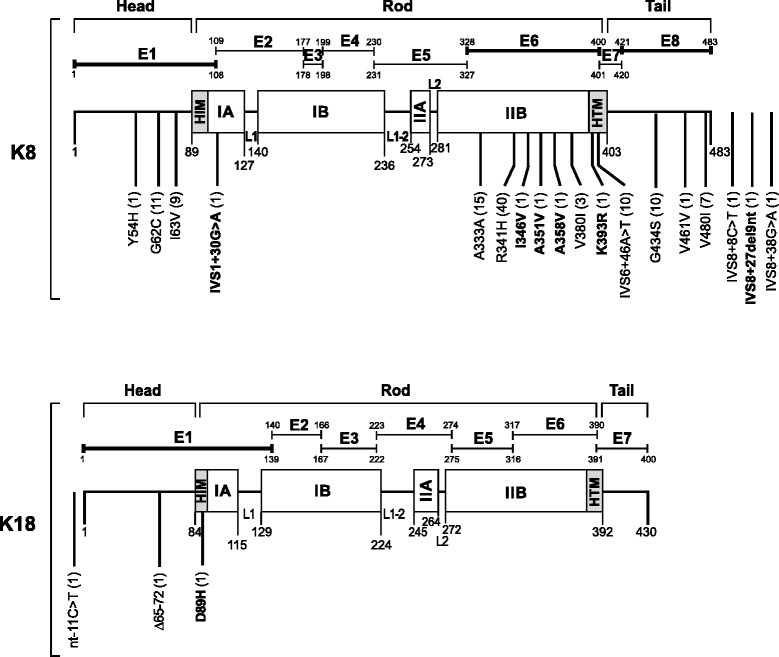
Distribution of the identified keratin 8/18 (K8/K18) variants within the protein backbone. The schematics depict the tripartite keratin structure consisting of head, rod and tail domains with their corresponding amino acid annotations. The rod subdomains IA, IB and II are connected by the corresponding linker (L) sequences, L1, L1-2, L2, and the shaded regions at the beginning and end of the rod domain correspond to particularly conserved helix initiation/termination motifs (HIM/HTM) that represent the mutational hotspots in epidermal keratins. The exonic structure of both genes is also depicted (E1–E8 for K8 and E1–E7 for K18). The exons that were analyzed in the present study are highlighted in bold. The coding variants are denoted by position and alteration of the affected amino acid displayed by the single-letter code. The relation of the intronic variants to the coding sequences is also shown. For annotation of intronic variants, the intervening sequences are labeled as ‘IVS’ and the position of a single variant located in the 5′UTR of K18 is denoted by its nucleotide (‘nt’) distance from the starting codon. The novel variants which were not previously described are highlighted in bold and the absolute count of all variants is listed in parentheses. Note that the novel K8 K393R and K18 D89H represent the first described K8/K18 variants localized in the most conserved HTM/HIM regions

The analysis of K8/K18 mutational hotspots revealed heterozygous exonic and intronic K8/K18 variants in 101 (12.6 %) and 15 (1.9 %) patients, respectively (Table 2). Amino acid-altering variants were found in 86 subjects (10.8 %; Table 2). K8 R341H constituted the most frequent amino acid-altering variant found in 40 subjects (5 %; Fig. 1) and was particularly common among Hispanics (9/88, 10.2 %; Table 2). On the other hand, K8 A333A (13/87, 14.9 %) and K8 G434S (10/87, 11.5 %) variants were the most frequent variants among African-Americans and the latter greatly contributed to the fact that this ethnicity displayed a high percentage of amino acid-altering variants (15/87, 17.2 %). K8 IVS6+46A>T represented the most common intronic variant that was seen in ten patients (1.3 %; Fig. 1). We also detected eight novel and hitherto undescribed variants including five amino acid-altering (K8 K393R, K8 A351V, K8 A358V, K8 I346V, K18 D89H; Fig. 1; Additional file 1: Figure S1). Four patients harbored compound heterozygous variants (K8 G62C+R341H, K8 R341H+K18-11C>T, K8 A333A+G434S, K8 I346V+IVS6+46A>T; Table 2).

**Table 2:** Distribution of keratin variants in DILI patients stratified by subject race/ethnicity

			Race/ethnicity					
			AA	Asian	Caucasian	Hispanic	Other	Total
			# (%)	# (%)	# (%)	# (%)	# (%)	# (%)
Nucleotide	Variant	dbSNP ID	87 (10.9)	25 (3.1)	577 (72.1)	88 (11.0)	23 (2.9)	800 (100)
160 T>C	K8 Y54H	rs57749775	0	0	0	1 (1.1)	0	1 (0.1)
184G>T	K8 G62C	rs11554495	0	0	10 (1.7)^c^	0	1 (4.3)	11 (1.4)
187A>G	K8 I63V	rs59536457	0	0	8 (1.4)	0	1 (4.3)	9 (1.1)
IVS1+30G>A^e^	K8 Intr	-	0	0	1 (0.2)	0	0	1 (0.1)
999C>T	K8 A333A	rs7750	13 (14.9)^a^	0	0	2 (2.3)	0	15 (1.9)
1022G>A	K8 R341H	rs57422427	1 (1.1)^b^	1 (4.0)	27 (4.7)^c^	9 (10.2)	2 (8.7)	40 (5.0)
1036A>G^e^	K8 I346V	-	0	0	1 (0.2)^d^	0	0	1 (0.1)
1052C>T^e^	K8 A351V	-	0	0	1 (0.2)	0	0	1 (0.1)
1073C>T^e^	K8 A358V	-	0	0	1 (0.2)	0	0	1 (0.1)
1138G>A	K8 V380I	rs56997521	0	0	3 (0.5)	0	0	3 (0.4)
1178A>G^e^	K8 K393R	-	0	0	1 (0.2)	0	0	1 (0.1)
IVS6+46A>T	K8 Intr	rs189690662	0	0	9 (1.6)^d^	0	1 (4.3)	10 (1.3)
1300G>A	K8 G434S	rs58573614	10 (11.5)^a^	0	0	0	0	10 (1.3)
1383G>T^e^	K8 V461V	-	0	1 (4.0)	0	0	0	1 (0.1)
1438G>A	K8 V480I	rs61730606	4 (4.6)	0	3 (0.5)	0	0	7 (0.9)
IVS8+8C>T	K8 Intr	rs201942002	0	0	0	1 (1.1)	0	1 (0.1)
IVS8+38G>A	K8 Intr	rs267607663	1 (1.1)	0	0	0	0	1 (0.1)
IVS8+27del9nt^e^	K8 Intr	-	0	0	1 (0.2)	0	0	1 (0.1)
K18-11C>T	K18 Intr	-	1 (1.1)^b^	0	0	0	0	1 (0.1)
K18 Δ65-72	K18 Deletion	rs267607417	0	0	0	1 (1.1)	0	1 (0.1)
265G>C^e^	K18 D89H	-	0	1 (4.0)	0	0	0	1 (0.1)
# (%) patients with amino acid-altering variants			15 (17.2)	2 (8.0)	54 (9.4)	11 (12.5)	4 (17.4)	86 (10.8)
# (%) patients with intronic variants			2 (2.3)	0	11 (1.9)	1 (1.1)	1 (4.3)	15 (1.9)

The table displays the number of patients of different races/ethnicities harboring the listed keratin variants. ^a^One patient carries two K8/K18 variants (K8 A333A+G434S); ^b^one patient carries two K8/K18 variants (K8 R341H+K18-11C>T); ^c^one patient carries two K8/K18 variants (K8 G62C+R341H); ^d^one patient carries two K8/K18 variants (K8 I346V+IVS6+46A>T); ^e^novel variants, which were not previously described (includes five amino acid-altering variants). AA, African-American; DILI, drug-induced liver injury

To test whether the detected K8/K18 variants are overrepresented among DILI subjects, we compared their frequencies in DILI patients with data from public databases. All common variants, as well as the total amino acid-altering K8/K18 variants, were found at comparable frequencies in DILI subjects and ethnically matched controls (Table 3); and similar results were obtained when only patients with high causality scores (of 1, 2, 3) were analyzed (not shown). As reported previously, several variants segregated with specific ethnicities. In particular, K8 G62C, I63V, R341H and V380I were overrepresented in Caucasians as compared to African-American controls; whereas K8 A333A, G434S and V480I were more common in African-Americans (Table 3). Although the data on Hispanic and Asian controls were rather limited, they demonstrated that K8 R341H is particularly common among Hispanics (that is, significantly more common than in both African-Americans and Caucasians), while African-American-enriched variants (K8 A333A and G434S) are rare in this group (Table 3).

**Table 3:** Distribution of selected keratin variants in the DILI and population control groups by subgroup

	Subgroup (#)		K8 G62C	K8 I63V	K8 A333A	K8 R341H	K8 V380I	K8 G434S	K8 V480I	Total^a^
		#	11/800	9/800	15/800	40/800	3/800	10/800	7/800	86/800
		%	1.4	1.1	1.9	5.0	0.4	1.3	0.9	10.8
DILI	Caucasian (577)	#	10^b^	8	0	27^b^	3	0	3	54
		%	1.7	1.4	0	4.7	0.5	0	0.5	9.4
	African-American (87)	#	0	0	13^c^	1	0	10^c^	4	15
		%	0	0	14.9	1.2	0	11.5	4.6	17.2
	Hispanic (88)	#	0	0	2	9	0	0	0	11
		%	0	0	2.3	10.2	0	0	0	12.5
	Asian (25)	#	0	0	0	1	0	0	0	2
		%	0	0	0	4.0	0	0	0	8.0
Control	Caucasian	#	59/4,296^1^	30/4,296^2^	8/4,298^3^	259/4,298^4,10^	7/4,297^5^	1/3,953^6^	6/3,980^7^	406/4,300
		%	1.4	0.7	0.2	6.0	0.2	0.03	0.2	9.4
	African-American	#	7/2,200^1^	3/2,201^2^	274/2,201^3,8,9^	35/2,203^4,11^	0/2,203^5^	169/2,075^6,12,13^	56/2,086^7^	388/2,203
		%	0.3	0.1	12.4	1.6	0	8.1	2.7	17.6
	Hispanic	#	-	1/121	1/121^8^	14/121^10,11^	-	0/121^12^	0/121	16/121
		%	-	0.8	0.8	11.6	-	0	0	13.2
	Asian	#	-	0/286	0/286^9^	7/286	-	0/286^13^	0/286	7/286
		%	-	0	0	2.4	-	0	0	2.4

Superscript numbers highlight comparisons between specific subgroups and the respective *P* values for these comparisons. For example, ^1^indicates a comparison of the frequency of the K8 G62C variant in Caucasian versus African-American controls. ^1^ *P* <0.0001; ^2^ *P* <0.002; ^3^ *P* <0.0001; ^4^ *P* <0.0001; ^5^ *P* = 0.1; ^6^ *P* <0.0001; ^7^ *P* <0.0001; ^8^ *P* <0.0001; ^9^ *P* <0.0001; ^10^ *P* <0.02; ^11^ *P* <0.0001; ^12^ *P* <0.0001; ^13^ *P* <0.0001. ^a^Amino acid-altering variants are summarized; ^b^one patient carries two K8/K18 variants (K8 G62C+R341H); ^c^one patient carries two K8/K18 variants (K8 A333A+G434S). DILI, drug-induced liver injury

The most frequently implicated agents in our cohort included amoxicillin-clavulanate, isoniazid and nitrofurantoin-induced DILI, and all these subgroups displayed similar distribution of K8/K18 variants (see Additional file 2: Table S1). Further analysis revealed that K8/K18 variants are distributed similarly among patients with different DILI causality or severity scores as well as injury patterns (Tables 4, 5 and 6). However, there is a trend of amino acid-altering keratin variants to associate with fatal/severe DILI (14 %) as compared with moderate/mild DILI (9.7 %; *P* = 0.09; Table 4) and this trend became more obvious when only patients with high causality scores were analyzed (14.3 % versus 8.7 %; *P* = 0.05).

**Table 4:** Distribution of keratin variants among DILI patients stratified by severity

Variant	Severity					
	Fatal (5)	Severe (4)	Mod hosp (3)	Mod (2)	Mild (1)	Total
	# (%)	# (%)	# (%)	# (%)	# (%)	# (%)
	71 (8.9)	144 (18.0)	221 (27.6)	176 (22.0)	188 (23.5)	800 (100)
K8 Y54H	0	0	0	0	1 (0.5)	1 (0.1)
K8 G62C	0	3 (2.1)	2 (0.9)	3 (1.7)	3 (1.6)^d^	11 (1.4)
K8 I63V	1 (1.4)	2 (1.4)	2 (0.9)	1 (0.6)	3 (1.6)	9 (1.1)
IVS1+30G>A	0	0	1 (0.5)	0	0	1 (0.1)
K8 A333A	2 (2.8)	4 (2.8)	5 (2.3)	1 (0.6)^c^	3 (1.6)	15 (1.9)
K8 R341H	5 (7.0)	5 (3.5)	13 (5.9)^b^	7 (4.0)	10 (5.3)^d^	40 (5.0)
K8 I346V	0	1 (0.7)^a^	0	0	0	1 (0.1)
K8 A351V	0	0	0	0	1 (0.5)	1 (0.1)
K8 A358V	0	1 (0.7)	0	0	0	1 (0.1)
K8 V380I	0	1 (0.7)	0	1 (0.6)	1 (0.5)	3 (0.4)
K8 K393R	1 (1.4)	0	0	0	0	1 (0.1)
IVS6+46A>T	0	3 (2.1)^a^	4 (1.8)	1 (0.6)	2 (1.1)	10 (1.3)
K8 G434S	1 (1.4)	4 (2.8)	2 (0.9)	2 (1.1)^c^	1 (0.5)	10 (1.3)
K8 V461V	0	0	0	1 (0.6)	0	1 (0.1)
K8 V480I	1 (1.4)	3 (2.1)	2 (0.9)	1 (0.6)	0	7 (0.9)
IVS8+8C>T	0	1 (0.7)	0	0	0	1 (0.1)
IVS8+38G>A	0	0	1 (0.5)	0	0	1 (0.1)
IVS8+27del9nt	0	0	1 (0.5)	0	0	1 (0.1)
K18-11C>T	0	0	1 (0.5)^b^	0	0	1 (0.1)
K18 Δ65-72	0	0	0	0	1 (0.5)	1 (0.1)
K18 D89H	1 (1.4)	0	0	0	0	1 (0.1)
# (%) patients with amino acid-altering variants	10 (14.1)	20 (13.9)	21 (9.5)	15 (8.5)	20 (10.6)	86 (10.8)
# (%) patients with intronic variants	0	4 (2.8)	8 (3.6)	1 (0.6)	2 (1.1)	15 (1.9)

^a^One patient carries two K8/K18 variants (K8 I346V+IVS6+46A>T); ^b^one patient carries two K8/K18 variants (K8 R341H+K18-11C>T); ^c^one patient carries two K8/K18 variants (K8 A333A+G434S); ^d^one patient carries two K8/K18 variants (K8 G62C+R341H); DILI, drug-induced liver injury; Mod, moderate non-hospitalized; Mod hosp, moderate hospitalized

**Table 5:** Distribution of keratin variants among DILI patients stratified by causality scores

Variant	Causality^e^					
	Definitive	Very likely	Probable	Possible	Unlikely	Total
	# (%)	# (%)	# (%)	# (%)	# (%)	# (%)
	190 (23.8)	312 (39.0)	148 (18.5)	110 (13.8)	39 (4.8)	800 (100)
K8 Y54H	0	0	0	1 (0.9)	0	1 (0.1)
K8 G62C	3 (1.6)	5 (1.6)^b^	2 (1.4)	1 (0.9)	0	11 (1.4)
K8 I63V	2 (1.1)	3 (1.0)	2 (1.4)	0	2 (5.1)	9 (1.1)
IVS1+30G>A	0	0	1 (0.7)	0	0	1 (0.1)
K8 A333A	3 (1.6)	2 (0.6)	3 (2.3)	4 (3.6)^d^	3 (7.7)	15 (1.9)
K8 R341H	8 (4.2)^a^	14 (4.5)^b^	8 (5.4)	9 (8.2)	1 (2.6)	40 (5.0)
K8 I346V	0	1 (0.3)^c^	0	0	0	1 (0.1)
K8 A351V	1 (0.5)	0	0	0	0	1 (0.1)
K8 A358V	1 (0.5)	0	0	0	0	1 (0.1)
K8 V380I	1 (0.5)	0	0	1 (0.9)	1 (2.6)	3 (0.4)
K8 K393R	0	0	1 (0.7)	0	0	1 (0.1)
IVS6+46A>T	2 (1.1)	3 (1.0)^c^	4 (2.7)	1 (0.9)	0	10 (1.3)
K8 G434S	2 (1.1)	2 (0.6)	2 (1.4)	4 (3.6)^d^	0	10 (1.3)
K8 V461V	0	0	0	0	1 (2.6)	1 (0.1)
K8 V480I	0	5 (1.6)	2 (1.4)	0	0	7 (0.9)
IVS8+8C>T	1 (0.5)	0	0	0	0	1 (0.1)
IVS8+38G>A	0	0	0	1 (0.9)	0	1 (0.1)
IVS8+27del9nt	0	0	0	1 (0.9)	0	1 (0.1)
K18 -11C>T	1 (0.5)^a^	0	0	0	0	1 (0.1)
K18 Δ65-72	0	1 (0.3)	0	0	0	1 (0.1)
K18 D89H	0	1 (0.3)	0	0	0	1 (0.1)
# (%) patients with amino acid-altering variants	18 (9.5)	31 (9.9)	17 (11.5)	16 (14.6)	4 (10.3)	86 (10.8)
# (%) patients with intronic variants	4 (2.1)	3 (1.0)	5 (3.4)	3 (2.7)	0	15 (1.9)

^a^One patient carries two K8/K18 variants (K8 R341H+K18-11C>T); ^b^one patient carries two K8/K18 variants (K8 G62C+R341H); ^c^one patient carries two K8/K18 variants (K8 I346V+IVS6+46A>T); ^d^one patient carries two K8/K18 variants (K8 A333A+G434S); ^e^the causality score was not known in one patient. DILI, drug-induced liver injury

**Table 6:** Distribution of keratin variants among DILI patients stratified by laboratory injury profile

Variant	Injury type			
	Hepatocellular	Cholestatic	Mixed	Total
	# (%)	# (%)	# (%)	# (%)
	438 (54.8)	198 (24.7)	164 (20.5)	800 (100)
K8 Y54H	0	1 (0.5)	0	1 (0.1)
K8 G62C	5 (1.1)	4 (2.0)^c^	2 (1.2)	11 (1.4)
K8 I63V	8 (1.8)	1 (0.5)	0	9 (1.1)
IVS1+30G>A	0	0	1 (0.6)	1 (0.1)
K8 A333A	4 (0.9)	7 (3.5)^d^	4 (2.4)	15 (1.9)
K8 R341H	21 (4.8)^a^	10 (5.0)^c^	9 (5.5)	40 (5.0)
K8 I346V	1 (0.2)^b^	0	0	1 (0.1)
K8 A351V	1 (0.2)	0	0	1 (0.1)
K8 A358V	0	1 (0.5)	0	1 (0.1)
K8 V380I	0	1 (0.5)	2 (1.2)	3 (0.4)
K8 K393R	0	1 (0.5)	0	1 (0.1)
IVS6+46A>T	7 (1.6)^b^	1 (0.5)	2 (1.2)	10 (1.3)
K8 G434S	5 (1.1)	5 (2.5)^d^	0	10 (1.3)
K8 V461V	1 (0.2)	0	0	1 (0.1)
K8 V480I	5 (1.1)	2 (1.0)	0	7 (0.9)
IVS8+8C>T	1 (0.2)	0	0	1 (0.1)
IVS8+38G>A	0	0	1 (0.6)	1 (0.1)
IVS8+27del9nt	1 (0.2)	0	0	1 (0.1)
K18 -11C>T	1 (0.2)^a^	0	0	1 (0.1)
K18 Δ65-72	1 (0.2)	0	0	1 (0.1)
K18 D89H	0	1 (0.5)	0	1 (0.1)
# (%) patients with amino acid-altering variants	46 (10.5)	27 (13.6)	13 (7.9)	86 (10.8)
# (%) patients with intronic variants	10 (2.3)	1 (0.5)	4 (2.4)	15 (1.9)

^a^One patient carries two K8/K18 variants (K8 R341H+K18-11C>T); ^b^one patient carries two K8/K18 variants (K8 I346V+IVS6+46A>T); ^c^one patient carries two K8/K18 variants (K8 G62C+R341H); ^d^one patient carries two K8/K18 variants (K8 A333A+G434S); DILI, drug-induced liver injury

**Fig. 2:**
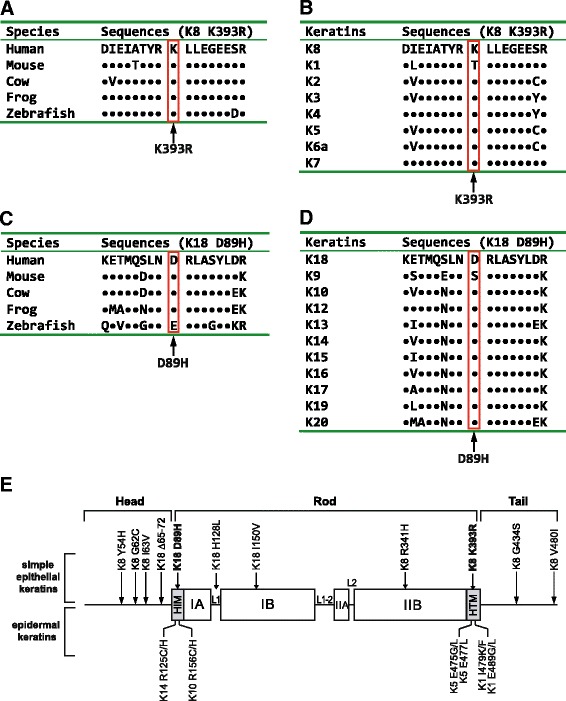
K8 K393R and K18 D89H are the first identified simple epithelial keratin variants located in the most conserved regions of the rod domain. Conservation of the novel K8 K393R/K18 D89H variants among (**a**,**c**) species and **b** type II or **d** type I keratins (standard single-letter amino acid abbreviations are used). Sequences surrounding the novel K8/K18 variants are depicted. Dots highlight conserved amino acids. **e** Distribution of simple epithelial and epidermal keratin variants within the keratin backbone. The schematic shows the characteristic tripartite structure (N-terminal ‘head’, central ‘rod’ and C-terminal ‘tail’ domains) of all IFs including keratins. The rod domain is subdivided into IA, IB, IIA, and IIB subdomains that in turn are separated through linker (L1, L1-2, L2) sequences. The shaded regions are the most conserved segments of the rod domain (termed helix initiation motif ‘HIM’ and helix termination motif ‘HTM’) and constitute mutation ‘hot spots’ in severe epidermal keratins and other IF mutations. Note that unlike epidermal keratins, the frequent human K8/K18 variants are located outside HIM/HTM. IF, intermediate filament

To better understand the biological significance of these newly identified K8/K18 variants, we studied their conservation by multiple sequence comparison. K8 K393R and K18 D89H variants were conserved both among species and type I and II keratins, respectively, which meshes well with the fact that they are located in the particularly conserved amino-terminal (K18 D89H, in an Asian patient with isoniazid-induced DILI) or carboxy-terminal regions (K8 K393R, in a Caucasian patient with ezetimibe and simvastatin-induced DILI) of the rod domain (Fig. 2) [2, 8]. Consistent with their biologically predicted importance due to amino acid conservation, both variants were found in patients with fatal DILI that otherwise constitutes <10 % of our cohort (Table 4). Both variants were observed in cholestatic DILI and were not detected among >300 Asian and >5,000 Caucasian controls analyzed so far, or available in the above described public databases (not shown). The K8 A358V variant was conserved among K8 from different species but not among other type II keratins and was detected in one subject with severe DILI (Table 4; Additional file 3: Figure S2). On the other hand, the non-conserved K8 A351V variant was observed in a participant with mild DILI.

### *In vitro* transfection experiments

**Fig. 3:**
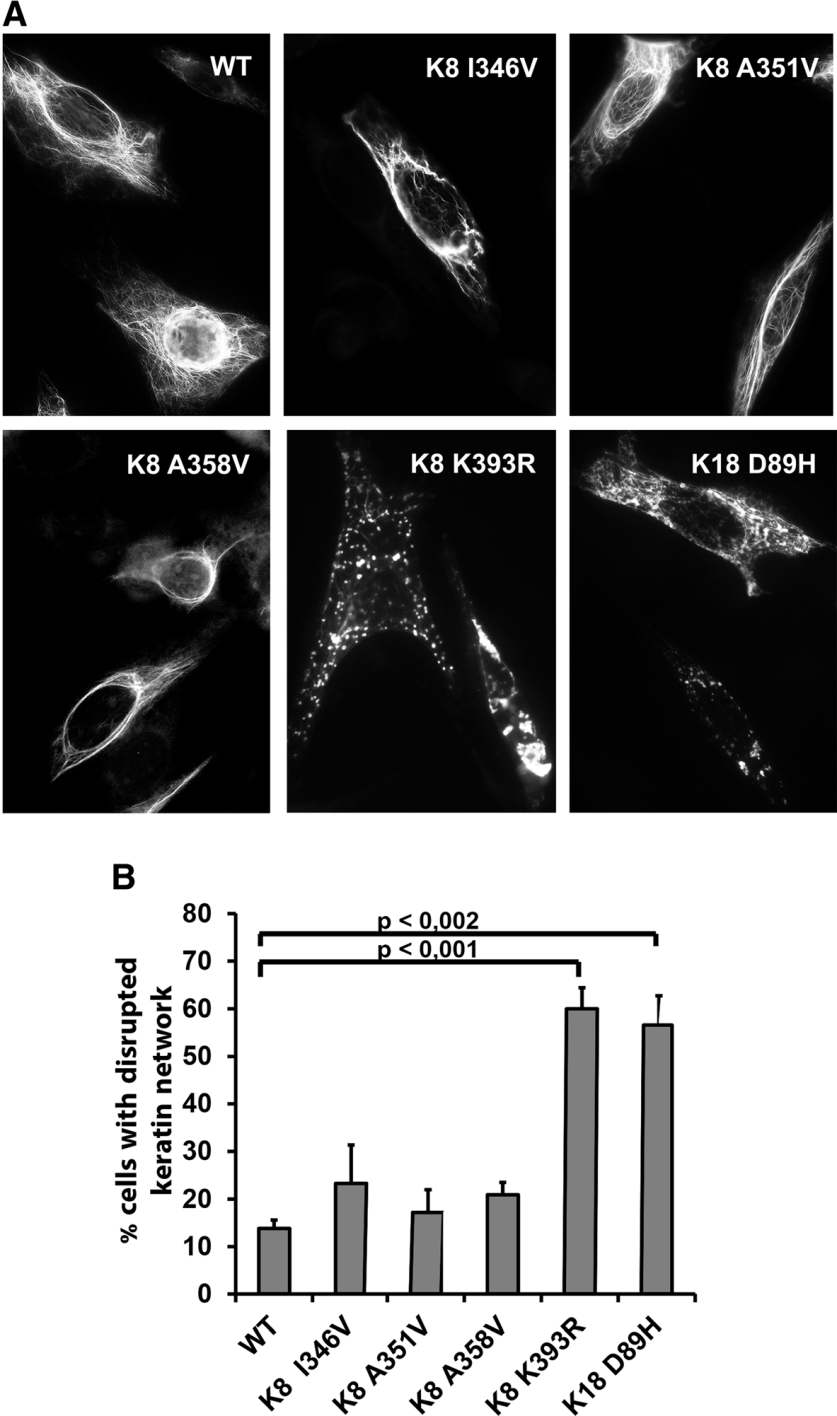
K8 K393R and K18 D89H variants result in keratin network disruption. **a** NIH 3T3 cells were transiently transfected with wild-type K8/K18 (WT) or a combination of K8/K18 variants and a WT partner keratin and stained with an anti-K18 antibody. Transfections were performed in triplicate. **b** The percentage of cells with disrupted keratin filaments was quantified. At least 100 cells were counted in each experiment

To address the impact of the novel K8/K18 variants on keratin filament network architecture, we performed transient transfections in NIH 3T3 cells (Fig. 3). Importantly, both K8 K393R and K18 D89H, but not the other K8/K18 variants, resulted in significantly more frequent disruption of the keratin filament network (Fig. 3). Collectively, these data suggest that rare K8/K18 variants which result in disruption of the keratin cytoskeleton are likely to predispose to severe DILI.

## Discussion

Our results demonstrate that keratin variants are not overrepresented in DILI patients, which contrasts with the data from patients with chronic hepatitis C, primary biliary cirrhosis and ALF [8, 19]. This lack of overall association of K8/K18 variants with DILI may, in part, be due to the limited number of cases attributed to an individual agent, since this cohort of 800 cases included over 250 individual drugs and herbal and dietary supplements. Along these lines, previous reports suggested that K8/K18 variants predispose only to specific forms of liver injury. For example, exonic variants were not found to predispose to disease development in patients with hemochromatosis [17], and transgenic animals carrying the K18 R90C variant were markedly susceptible to Fas- but not TNFα-induced apoptosis as well as to thioacetamide- but not carbon tetrachloride-induced liver fibrosis [32].

To evaluate the importance of K8/K18 variants in DILI, we took advantage of large, publicly available databases that allowed us to compare our results with a larger control population than the previous studies [8, 19]. Multiple K8/K18 variants were confirmed and shown to associate with specific ethnic backgrounds [19, 30] and R341H has been demonstrated to constitute the most common amino acid-altering K8/K18 variant in Asians (Table 3). In addition, K8 A333A, G434S and V480I were largely restricted to African-Americans as compared to both Asians and Hispanics. While the exact reason for the enrichment of certain K8/K18 variants in specific ethnic backgrounds remains unknown, unequal ethnic distribution of human variants is relatively common and is caused by adaption to local factors [33, 34].

The data from publicly available databases indicate that the frequency of several common variants is higher than previous estimations. In particular, K8 G62C and R341H variants were detected in 1.4 % and 6 % of US Caucasian controls, respectively (versus 0.9 % and 3.2 % as reported previously [19, 35]). These data should therefore inform future studies that examine K8/K18 variants in different disease contexts.

While we found no overrepresentation of common K8/K18 variants in DILI subjects, we observed a clear trend towards clustering of these variants in the more severe cases. However, due to the relatively low numbers, further studies are needed to confirm this observation that is reminiscent of the situation with ALF, where the presence of K8/K18 variants was associated with adverse outcomes [19]. Moreover, we detected several previously unknown variants that are likely to have biological importance. As such, K8 K393R and K18 D89H are the first described simple epithelial keratin variants located in the most conserved helix initiation/termination motifs (HIM/HTM) of the rod domain (Fig. 2e). Of note, mutations in these domains have been presumed to be embryolethal in humans [19, 35, 36] and represent the most important and abundant disease-causing mutational hotspots in epidermal keratins and other IFs (Fig. 2e) [2, 8, 13, 37]. The observed keratin network disruption in cells transfected with K8 K393R or K18 D89H further strengthens their biological significance. Although the performed transfection experiments cannot fully evaluate the importance of both variants *in vivo*, the conserved K18 D89H represents a non-conservative substitution since it alters protein charge. Moreover, it is only one residue away from K18 R90C (Fig. 2) whose overexpression in transgenic mice causes a marked susceptibility to a variety of drug-induced liver injuries [8, 35]. While K8 K393R might be considered a conservative substitution, the same mutation in a less conserved region of K5 (that is, K5 K199R) was sufficient to cause EBS [38]. Hence, structural considerations, cell culture experiments, transgenic mouse data and the fact that both patients with K8 K393R/K18 D89H variants suffered fatal DILI collectively make a strong case for the importance of these substitutions in DILI outcomes.

Although the exact pathogenesis of DILI remains to be clearly defined [22], K8/K18 deficiency results in multiple cellular dysfunctions that likely contribute to development/progression of this type of liver injury. For example, keratins constitute important mechanoprotective proteins and K18 R90C mutation results in hepatocyte fragility, while other tested keratin mutations that are not in the HIM/HTM do not [35]. Furthermore, K8/K18 are established anti-apoptotic genes [8] and may also protect from hepatocyte necrosis as demonstrated in transgenic mice that express K18 D238E/D397E which cannot be cleaved by caspases during apoptosis [39]. Keratins are also known to modulate organelle positioning, and impairment in K8/K18 result in mitochondrial dysfunction and susceptibility to oxidative stress [40, 41]. Furthermore, keratins act as signalling platforms and are important for protein localization/targeting, modulation of protein synthesis, cell growth or proliferation [3, 8, 42–44]. Although the commonly found K8 variants do not result in obvious keratin network redistribution under basal condition, they do so under various stress situations such as oxidative stress or upon hyperphosphorylation conditions [45]. In that respect, K8 G62C/R341H/R341C variants predisposed transgenic mice to acetaminophen-induced liver injury and this predisposition was associated with a more prominent keratin network disruption after the exposure to the drug [46].

Fortunately severe clinical outcomes with DILI are uncommon, with less than 10 % of patients requiring a liver transplant or dying within 6 months of DILI onset [47]. However, additional genetic studies that focus on the HIM/HTM domains of simple epithelial keratins are likely to provide important information regarding the prevalence of fatal DILI in individuals with HIM/HTM keratin variants. We also anticipate that specific drugs, and potentially specific race and ethnic background variants, are likely to play important roles in this process.

## Conclusions

Our study has uncovered variants in highly conserved residues of K8 Lys393Arg and K18 Asp89His in patients with fatal DILI. These first to be described novel variants represent for the first time described ‘epidermal-like’ K8/K18 variants which lead to keratin network disruption in untreated cells. Common K8/K18 variants were found at similar frequencies in DILI subjects and ethnically matched population controls. Thus, rare K8/K18 variants may predispose to DILI development in a subset of patients.

## Additional files

Additional file 1: Figure S1. Identification of the novel variants (**A**) K8 K393R and (**B**) K18 D89H. A comparison with wild type (WT) sequences (left panels) reveals the heterozygous nature of the depicted variants. Standard single-letter amino acid abbreviations are used. (PDF 2587 kb) Additional file 2: Table S1. Distribution of K8 and K18 variants in selected drug categories. (DOCX 44 kb) Additional file 3: Figure S2. Conservation of the novel K8 variants among (**A**,**C**,**E**) species and among (**B**,**D**,**F**) type II keratins. Sequences surrounding the novel K8 variants are depicted. Dots highlight conserved amino acids (listed using standard single-letter abbreviations). (PDF 530 kb)

## Abbreviations

AA: African-American
ALF: Acute liver failure
DILI: Drug-induced liver injury
DILIN: DILI Network
DMEM: Dulbecco’s modified Eagle’s medium
EBS: Epidermolysis bullosa simplex
EDTA: Ethylenediaminetetraacetic acid
FCS: Fetal calf serum
HIM: Helix initiation motif
HPLC: High-performance liquid chromatography
HTM: Helix termination motif
IF: Intermediate filament
K: Keratin
K8: Keratin 8
K18: Keratin 18
PBS: Phosphate-buffered saline
PCR: Polymerase chain reaction
