# Supplementary figures and images for: Prevalence of genetic variants of keratins 8 and 18 in patients with drug-induced liver injury
Source: BMC Med. 2015 Aug 19;13:196. doi: 10.1186/s12916-015-0418-0 (PMC4545365; doi:10.1186/s12916-015-0418-0)

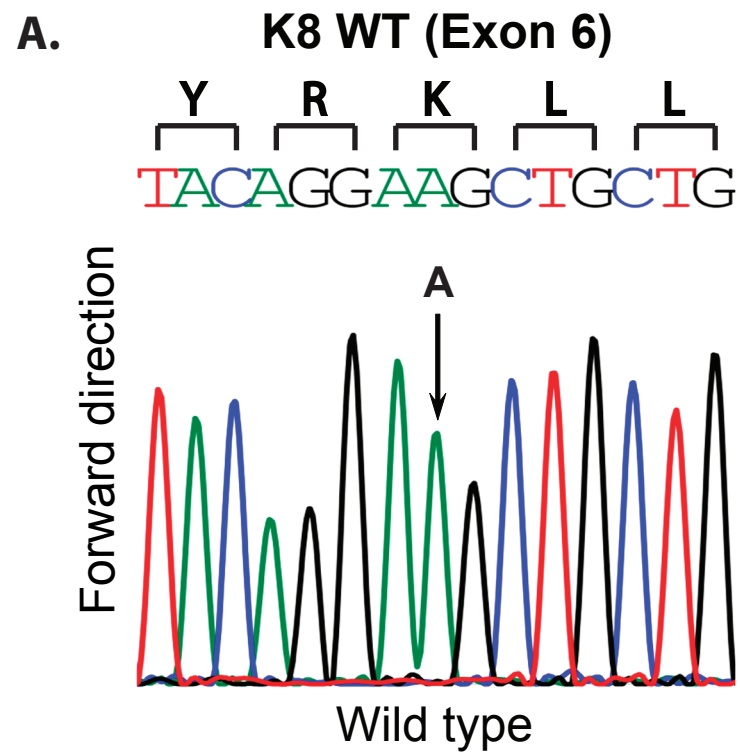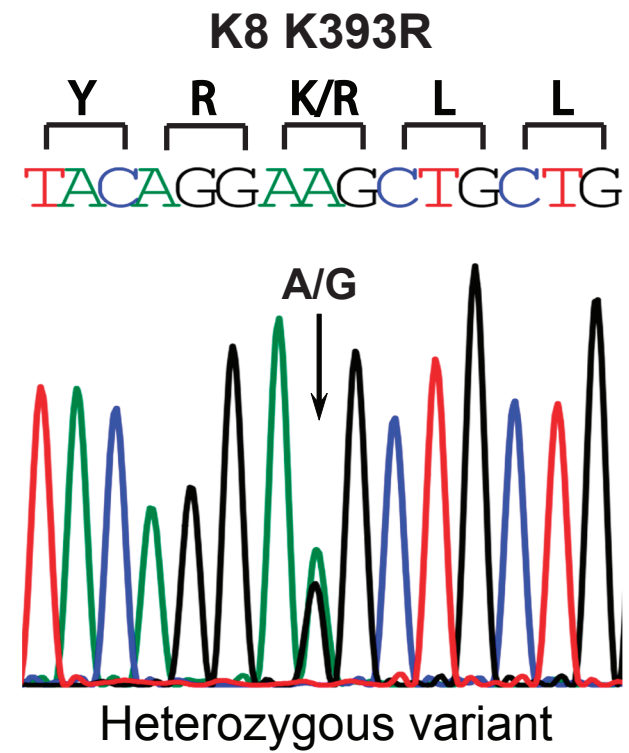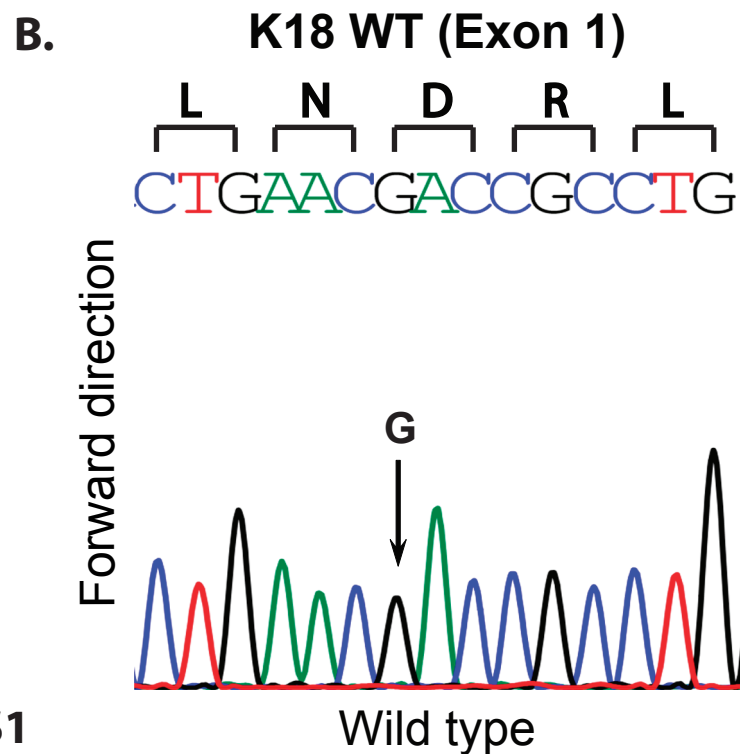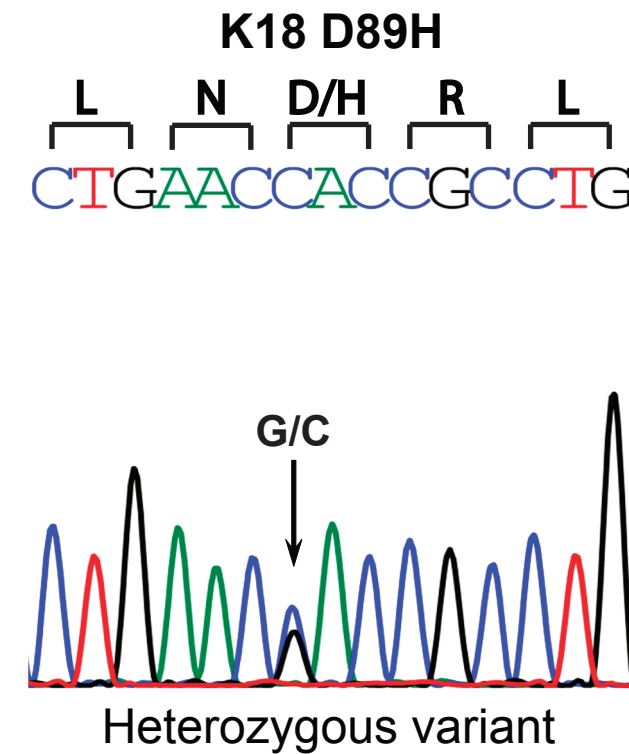

Supplement: Additional file 1: Figure S1. — Identification of the novel variants (A) K8 K393R and (B) K18 D89H. A comparison with wild type (WT) sequences (left panels) reveals the heterozygous nature of the depicted variants. Standard single-letter amino acid abbreviations are used. (PDF 2587 kb) [file 12916_2015_418_MOESM1_ESM.pdf]
