# Supplementary material for: Prevalence of genetic variants of keratins 8 and 18 in patients with drug-induced liver injury
Source: BMC Med. 2015 Aug 19;13:196. doi: 10.1186/s12916-015-0418-0 (PMC4545365; doi:10.1186/s12916-015-0418-0)
Supplement: Additional file 2: Table S1. — Distribution of K8 and K18 variants in selected drug categories. (DOCX 44 kb) [file 12916_2015_418_MOESM2_ESM.docx]

**Additional file 2: Table S1 - Distribution of K8 and K18 variants in selected drug categories**

| **Drug** | **# patients with amino acid-altering variants / # patients** | **% patients with amino acid-altering variants** | **Identified Variants**¶ |
| --- | --- | --- | --- |
| Amoxicillin | 10/93 | 10.8 | 1 K8 I63V, 7 K8 R341H,  2 K8 G434S |
| Isoniazid | 7/51 | 13.7 | 2 K8 I63V, 2 K8 R341H,  1 K8 G434S, 1 K8 V480I,  1 K18 D89H |
| Nitrofurantoin | 4/41 | 9.8 | 1 K8 G62C, 1 K8 I63V,  1 K8 R341H, 1 K8 V380I |
| SulfamethoxazoleW/  Trimethoprim | 4/32 | 12.5 | 1 K8 G62C, 1 K8 I63V,  1 K8 R341H, 1 K8 I346V |
| All Other Therapeutic Products | 4/23 | 17.4 | 3 K8 R341H, 1 K8 I63V |
| Valproic Acid | 2/23 | 8.7 | 1 K8 R341H, 1 K8 A351V |
| Unspecified Herbal | 4/21 | 19.1 | 1 K8 G62C, 1 K8 R341H,  2 K8 V480I |
| Other Combinations Of Nutrients | 5/11 | 45.5 | 1 K8 I63V, 2 K8 R341H,  2 K8 G434S |
| Duloxetine | 2/8 | 25.0 | 1 K8 G62C, 1 K8 A358V |
| Methyldopa | 2/7 | 28.6 | 1 K8 G62C, 1 K8 G434S |
| Simvastatin | 2/7 | 28.6 | 2 K8 R341H |
| Hydralazine | 2/4 | 50 | 1 K8 G62C^1^, 2 K8 R341H^1^ |
| Total | 48/321 | 15.0 |  |

^1^One patient carries two K8 variants (K8 G62C+R341H).
