# Supplementary material for: Prevalence of genetic variants of keratins 8 and 18 in patients with drug-induced liver injury
Source: BMC Med. 2015 Aug 19;13:196. doi: 10.1186/s12916-015-0418-0 (PMC4545365; doi:10.1186/s12916-015-0418-0)
Supplement: Additional file 3: Figure S2. — Conservation of the novel K8 variants among (A,C,E) species and among (B,D,F) type II keratins. Sequences surrounding the novel K8 variants are depicted. Dots highlight conserved amino acids (listed using standard single-letter abbreviations). (PDF 530 kb) [file 12916_2015_418_MOESM3_ESM.pdf]

A.

Species	Sequences (K8 A351V)		
Human	ELAIKDAN	A	KLSELEAA
Mouse	•M•••••Q	T	••A•••••
Cow	•M•V•••Q	•	••A•••••
Frog	•••L•••R	N	••A•••••
Zebrafish	•M•VR••K	G	RIKD••D•

A351V

B.

Keratins	Sequences (K8 A351V)		
K8	ELAIKDAN	A	KLSELEAA
K1	•N•L•••K	N	••ND••D•
K2	•H•L•••R	N	••ND••E•
K3	•M•L••••	•	••Q••Q••
K4	•N•L•••H	S	•RV•••••
K5	•L•L•••R	N	••A•••E•
K6a	•M•L•••K	N	••EG••D•
K7	•••L•••R	•	•QE•••••

A351V

C.

Species	Sequences (K8 A358V)		
Human	NAKLSELE	A	ALQRAKQD
Mouse	QT••A•••	•	••••••••
Cow	Q•••A•••	•	••RN••••
Frog	RN••A•••	•	•••K••••
Zebrafish	KGRIKD••	D	••••••••

A358V

D.

Keratins	Sequences (K8 A358V)		
K8	NAKLSELE	A	ALQRAKQD
K1	KN••ND••	D	•••Q••E•
K2	RN••ND••	E	•••Q••E•
K3	••••Q••Q	•	•••Q••D•
K4	HS•RV•••	•	•••Q••EE
K5	RN••A•••	E	•••K••••
K6a	KN••EG••	D	•••K••••
K7	R••QE••v	•	••••G•••

A358V

E.

Species	Sequences (K8 I346V)		
Human	AEQRGELA	I	KDANAKLS
Mouse	••••••MA	•	•••QT••A
Cow	••••••MA	V	•••Q•••A
Frog	••E••••A	L	•••RN••A
Zebrafish	••E•••MA	V	RD•KGRIK

I346V

F.

Keratins	Sequences (K8 I346V)		
K8	AEQRGELA	I	KDANAKLS
K1	••••••N•	L	•••KN••N
K2	••••••H•	L	•••RN••N
K3	•••H••M•	L	•••••••Q
K4	••••••N•	L	•••HS•RV
K5	••••••••	L	•••RN••A
K6a	••••••M•	L	•••KN••E
K7	••E•••••	L	•••R••QE

I346V

Additional file 3: Figure S2
